# Clinical Lectures on Surgery

**Published:** 1856-01

**Authors:** 


*Clinical Lectures on Surgery:* By M. NELATON, from notes taken by Walter F. Atlee, M. D. Philadelphia: J. B. Lippincott & Co., 1855.

As an exponent of the practice of M. Nelaton, this work will be read with interest by the Profession in this country. The notes were taken during the years 1851–52–53–54—and may be regarded as a fair expose of Prisian surgery, at least so far as it goes.

We have marked a few passages. M. Nelaton treats fractures of the patella by applying bandages soaked in plaster, taking care that the parts are previously in apposition, and retaining them there by pressure till the plaster hardens. In a case reported, bony union was believed to have taken place.

In a case of ununited fracture of the humerus, of four years standing, we find the following remarks of M. Nelaton, together with his treatment and its results :

" A great number of means have been proposed for the purpose of curing false joints. Here, it was plain that the simple application of an apparatus to keep the parts at rest would not be sufficient. The ancient method, employed by Celsus, was to rub the fragments roughly, one against the other ; but this is very little used, and it might be asked whether the cure would not have taken place just as well without it. The seton, passed at the seat of the fracture, is a method against which a great deal has been said. There is, however, no comparison to be made between the dangers of its employment in one limb, with those of another; in the femur, for instance, it is very dangerous. Here the conditions were simple, and it could be done very easily. In case that it was found that the seton could not be passed between the fragments, it would be necessary to pass two of them, one on each side.

The seton was passed, without difficulty, between the two extremities of the bone, at the seat of fracture. Desirous of exciting a good deal of inflammation, the next day a larger seton was drawn in, and the same thing was repeated the day after. A good deal of pain, and considerable inflammation, followed their introduction. The seton was allowed to remain for two weeks, and being then withdrawn, the arm was placed in an apparatus to keep it perfectly at rest.

The man did not remain a sufficient time in the wards to complete his cure there; but I have been assured that the parts became perfectly consolidated."

We make the following extract, giving the history of the treatment of a case of ranula:

"November, 1852. A man, about forty years of age, entered the wards for ranula. The history of the treatment he had already undergone was given, in order to show the difficulty there sometimes is in treating these tumors. In the summer of 1849, he went to M. Jobert, on account of a tumor situated under the tongue; they were about to operate upon him, when the tumor opened of itself. In the month of October, the tumor had returned, and M. Boyer, at the Hotel Dieu, cut it out. Early in 1850, the swelling under the tongue, had again returned, and he went a second time to M. Boyer, who again excised it; this time the operation was upon the left side, at the other, it was upon the right. In October of the same year, it had again returned, and M. Boyer made, this time, two punctures into it, one under the tongue, and the other externally under the jaw, for the tumor commenced to show itself there. These punctures only relieved him for a short time, and in January, 1851, he went to M. Thierroy, who passed a leaden wire into he tumor, under the jaw, and left it in that place for eight days; this produced nothing satisfactory. In November, the same surgeon placed a seton in the interior of the mouth, and afterward he made a third operation, in which a seton was passed inside of the mouth, through the tumor, and made to come out under the jaw. This last operation was followed by great inflammation, and M. Thierry being sick, M. Velpeau attended to the case; he made an incision two inches in length, into the large swelling, and the patient was relieved. M. Thierry afterward applied the instrument of Dupuytren, which resembles a shirt-button with a canal through the center. This history shows how much had been done to cure one of these tumors, and how it persisted in spite of all.

In order to cure the patient, the whole of the sac, the buccal and the submaxillary portions, would have to be obliterated. For this purpose, injections of iodine would be made use of. The membrane lining the whole of the interior of the cyst being lined by a thick viscous liquid, it must be thoroughly washed, without which the injection of iodine does not come in contact with the parts upon which it is to act.

The puncture was made with a bistoury, and, after washing the sac clean of its viscous contents, the injection was made. At the end of twenty-four hours, the tumor was reproduced; it had the same volume as before, was a little harder, and painful to the touch. Nothing more was done; things were allowed to have their course. On the fifth day, a commencement of absorption was evident in the swelling, which went on more rapidly than is usual after such injections, and the patient finally left, cured of his affection."

We have not space for further extracts. The Notes are concise, but sufficiently full to give us a clear and distinct idea of the cases and their tretament.

For sale by S. C. Griggs & Co. J.

